# SIRT6 Prevents Glucocorticoid-Induced Osteonecrosis of the Femoral Head in Rats

**DOI:** 10.1155/2022/6360133

**Published:** 2022-10-13

**Authors:** Lun Fang, Gang Zhang, Yadi Wu, Zhongzhe Li, Shan Gao, Lu Zhou

**Affiliations:** ^1^Institute of Sports Medicine, Shandong First Medical University & Shandong Academy Medical Sciences, Taian, 271016 Shandong Province, China; ^2^Department of Orthopedics, The Second Affiliated Hospital of Shandong First Medical University, Taian, 271000 Shandong Province, China; ^3^School of Pharmaceutical Science, Shandong First Medical University & Shandong Academy Medical Sciences, Taian, 271016 Shandong Province, China

## Abstract

**Objective:**

Glucocorticoid-induced osteonecrosis of the femoral head is one of the most common causes of nontraumatic osteonecrosis of the femoral head, but its exact pathogenesis remains unclear. The aim of this study was to investigate the role of SIRT6 in the maintenance of bone tissue morphology and structure, intravascular lipid metabolism, and its potential molecular mechanism in glucocorticoid-induced osteonecrosis of the femoral head.

**Methods:**

SIRT6 adenovirus was transfected into GIONFH in rats. The microstructure of rat bone was observed by micro-CT and histological staining, and the expression of bone formation-related proteins and angiogenesis-related factors was determined through western blot and immunohistochemistry. Alkaline phosphatase activity, alizarin red staining, and the expression levels of Runx2 and osteocalcin were used to evaluate the osteogenic potential. And *in vitro* tube formation assay and immunofluorescence were used to detect the ability of endothelial cell angiogenesis.

**Results:**

Dexamethasone significantly inhibited osteoblast differentiation, affected bone formation, and destroyed microvessel formation, increased the intracellular Fe^2+^ and ROS levels and induced the occurrence of ferroptosis. SIRT6 can inhibit ferroptosis and restore the ability of bone formation and angiogenesis.

**Conclusion:**

SIRT6 can inhibit the occurrence of ferroptosis, reduce the damage of vascular endothelium, and promote osteogenic differentiation, so as to prevent the occurrence of osteonecrosis of the femoral head.

## 1. Introduction

Nontraumatic osteonecrosis of the femoral head, a disease characterized by chronic pain and limited mobility of the hip, is the most common reason of hip replacement in young adults [1, 2]. Glucocorticoid-induced osteonecrosis of the femoral head (GIONFH) is caused by the massive use of glucocorticoids, and its prevalence accounts for the first place of nontraumatic osteonecrosis of the femoral head. The massive use of glucocorticoids will cause many pathophysiological changes in vivo, such as metabolic disorders, elevated blood glucose, and coagulation dysfunction, so that the blood is in a hypercoagulable state, which makes the local formation of thrombosis in the femoral head, leading to ischemic osteonecrosis of the femoral head [3, 4]. The excessive use of hormones leads to the decrease of osteogenic differentiation and the increase of lipogenic differentiation, thus blocking the blood supply of the femoral head, leading to the collapse of local bone tissue, which is the main factor leading to its necrosis [5]. According to previous studies, the pathogenesis of GIONFH can be summarized in two aspects: (1) impaired blood supply of femoral head and (2) diminished osteogenic activity. And some studies have found that strengthening angiogenesis and osteogenesis is the key to prevent GIONFH or provide early treatment of GIONFH [6].

Silent information regulatory protein (Sirtuin) is an evolutionary conserved protein that regulates key physiological processes such as apoptosis, metabolism, energy balance, mitochondrial function, and aging [7]. SIRT6, as a member of the Sirtuin family, has both NAD^+^-dependent histone deacetylase activity and ADP-ribosyltransferase activity [8]. SIRT6 has deacetylase activity against histone H3K9Ac and acts as a transcription factor hypoxia-inducible factor 1*α*, c-Jun, and nuclear factor *κ*B (NF-*Κ*b), deacetylating histone H3K9Ac at its target promoter and downregulating the expression of its target genes [9]. With the development of research, more and more biological functions of SIRT6 have been discovered, including delaying cellular senescence, regulating lipid metabolism, antirheumatism, and reversing myocardial hypertrophy. However, there is no study on the protection of vascular endothelial cells by SIRT6 to prevent or treat related vascular injurious necrosis diseases at home and abroad.

SIRT6 plays an important role in osteogenic differentiation and angiogenesis. This study was aimed at investigating the role of SIRT6 in the maintenance of bone tissue morphological structure, intravascular lipid metabolism, and its potential molecular mechanism in GIONFH, providing a theoretical basis for the clinical treatment of GIONFH.

## 2. Materials and Methods

### 2.1. Animals and GIONFH Models

A total of 75 male SD rats were randomly divided into the following five groups: normal control group (NC), hypoxia model control group (con), adenoviral function control group (LacZ), SIRT6 overexpression group (Ad-SIRT6), and SIRT6 mutation group (Mt-SIRT6). The femoral head necrosis model was created by intramuscular injection of 10 mg/kg dexamethasone sodium phosphate once every three days for 8 weeks; the blank control group was injected with saline [10]. LacZ, SIRT6, and SIRT6 mutant adenoviruses were injected into the tail vein of rats in the LacZ group and SIRT6 mutation group on the day before the start of modeling, respectively, to increase or decrease the corresponding protein levels. Adenovirus was used to increase the protein content of the SIRT6 gene, and LacZ virus was used as a functional control virus of adenovirus to detect the safety and identify adenovirus. SIRT6 mutant viruses do not possess deacetylase activity and only retain ADP-ribosyltransferase activity [11].

### 2.2. Microcomputed Tomography (Micro-CT)

When the GIONFH model was completed in SD rats, the femurs were collected from the rats immediately after euthanasia. The structure of the femoral head and cortical area was examined by microcomputed tomography. The following parameters were measured for the assessment of bone quality: trabecular bone volume/total volume (BV/TV), trabecular thickness (Tb. Th, mm), trabecular number (Tb. N, 1/mm), and trabecular separation (Tb. Sp, mm).

### 2.3. Histological Staining

Femurs were fixed in 10% buffered formalin and decalcified using 10% EDTA decalcification solution (pH 7.2–7.4) for 4 weeks, and fresh decalcification solution was replaced once every two days. After decalcification was completed and embedded in paraffin, the tissues were sectioned longitudinally [12]. Hematoxylin-eosin (HE) staining and safranin-O staining were then performed.

### 2.4. Immunohistochemical Staining

DAKO EnVision System (DAKO, Denmark) was used to perform Immunohistochemical staining, which uses horseradish peroxidase-conjugated dextran polymers. Then, the paraffin samples were warmed for 30 min at 55°C. Following deparaffinization, tissue sections were treated with in 0.01 M sodium citrate buffer (pH 6.0) and immunostained with CD31 and VEGF antibody using a microwave antigen retrieval procedure.

### 2.5. Cell Culture

Mouse preosteoblastic MC3T3-E1 cells (ATCC company) and human umbilical vein vascular endothelial cells HUVEC (Otwo Biotech (Shenzhen) Inc.) were used for the study. They were cultured with DMEM high-glucose medium containing 10% FBS, in which 100 *μ*g/ml streptomycin and 100 U/ml penicillin (Lot. P1400; Solarbio) were added and placed in an incubator with 5% CO_2_ and at 37°C.

### 2.6. Cell Model

To mimic culturing cells under ischemic conditions, we placed the cell culture in an Oxoid anaerobic tank with an Oxoid AnaeroGen anaerobic packet (cat.no. HBYY001; hopebio). The anaerobic bacterial sac in the sealed tank rapidly absorbs the oxygen in the atmosphere and produces CO_2_, and the oxygen concentration decreases to less than 1% within 1 h.

### 2.7. Cell Transfection and Grouping

Logarithmically growing MC3T3-E1 and HUVEC cells were seeded into 6-well plates and transfected when the cells were confluent to 30%–50%. LacZ adenovirus, SIRT6 adenovirus, and SIRT6 gene mutant adenovirus were added to serum-free and antibiotic-free medium, and fresh complete medium was replaced after 6 hours of transfection. Untreated cells were also used as the normal control group. After 24 hours of culture, the expression of SIRT6 in the cells was detected by western blot.

### 2.8. Alkaline Phosphatase (ALP) Staining and Alizarin Red (ARS) Staining

MC3T3-E1 cells were seeded in 6-well plates. When the cells reached 80% confluence, hypoxia treatment was performed, osteogenic induction medium (complete medium containing 10 mM *β*-sodium glycerophosphate (cat.no. A56289; OKA)) and 50 *μ*g/ml ascorbic acid were added to induce differentiation into mature osteoblasts [13], and fresh osteogenic induction medium was replaced every other day. The activity of alkaline phosphatase was measured by ALP staining kit (cat.no. D001-2; Jiancheng Biochemical) after 7 days' induction. Osteogenic differentiation was induced 21 days, and the mineralized nodule formation of MC3T3-E1 was determined using alizarin red solution (cat.no. C1452; Solarbio). Then, they were stained with 1% ARS for 5 min at 37°C. The cells were then washed with distilled water to remove excess dye and finally placed under a light microscope to observe and photograph the stained matrix.

### 2.9. Tube Formation *In Vitro*

Matrigel (cat.no. 0827045, ABW) was diluted with an equal volume of DMEM and added to a 96-well plate for plating, 50 *μ*l of Matrigel was added to each well, and the cells were seeded in a 96-well plate. HUVEC cells were observed for the formation of tube-like structures under a microscope.

### 2.10. Immunofluorescence

Cells were fixed with 4% paraformaldehyde for 30 min and blocked with 3% BSA for 30 min. Anti-VEGF antibody dilutions were incubated overnight. PBS was washed three times, and secondary antibodies were incubated for 1 h in the dark. DAPI was stained in the nucleus for 5 min. They were observed and photographed under a fluorescence microscope.

### 2.11. Assay of Ferrous Iron

Cells were collected and disrupted with ultrasonic waves. Centrifuge at 12,000 rpm for 10 min at 4°C, and take the supernatant. Add 1,10-phenanthroline (cat.no. 320056; Sigma-Aldrich), mix well, and place for 60 min. The absorbance was measured at 506 nm.

### 2.12. Determination of Glutathione (GSH)

The cells were collected by centrifugation, and the samples were subjected to repeated rapid freezing and thawing, and the supernatant was used for the determination of GSH. Intracellular total GSH levels were measured following the operating instructions of the GSH assay kit (cat.no. S0055; Beyotime).

### 2.13. Determination of Malondialdehyde (MDA)

MDA content was measured by MDA assay kit (cat.no. S0131S; Beyotime). Cells were disrupted by ultrasound, and the supernatant was taken. The collected supernatant was added to thiobarbituric acid. The absorbance was read at 532 nm. The MDA level indicates the ratio to the absorbance value of the normal control group.

### 2.14. Reactive Oxygen Species (ROS) Assay

ROS level was measured using Dihydroethidium (cat.no. S0063; Beyotime). Cells were seeded in 6-well plates and transfected. After 12 hours of hypoxia, 10 *μ*M Dihydroethidium was added and incubated with an incubator for 30 min, and the mean fluorescence intensity was measured by flow cytometry.

### 2.15. Western Blot

Total protein was extracted from the femoral heads and cells of each group with RIPA lysate, and protein concentration was determined with Coomassie brilliant blue. Protein samples of 30 *μ*g were transferred to PVDF (Polyvinylidene Difluoride) membranes after polyacrylamide gel electrophoresis. 5% skimmed milk was blocked for 1 h at room temperature, incubated overnight with primary antibodies for SIRT6 (1 : 1000; cat.no. 12486; Cell Signaling Technology, Inc.), HIF-1*α* (1 : 1000; cat.no. 14179; Cell Signaling Technology, Inc.), Acetyl-H3K9 (1 : 1000; cat.no. ab10812; Abcam), Runx2 (1 : 1000; cat.no. 12556; Cell Signaling Technology, Inc.), ALP (1 : 1000; cat.no. ab229126; Abcam), osteocalcin (OCN; 1 : 1000; cat.no. ab93876; Abcam), CD31 (1 : 1000; cat.no. 3528; Cell Signaling Technology, Inc.), VEGF (1 : 1000; cat.no. 9698; Cell Signaling Technology, Inc.), transferrin receptor (TFRC; 1 : 1000; cat.no. ab214039; Abcam), divalent metal transporter-1 (DMT1; 1 : 1000; cat.no. ab55735; Abcam), SLC7A11 (1 : 1000; cat.no. ab175186; Abcam), GPX4 (1 : 1000; cat.no. 59735; Cell Signaling Technology, Inc.), and *β*-actin (1 : 5000; cat.no. AB0035; Abways Technology), and the next day, secondary antibody was added and incubated for 1 h at room temperature for development. *β*-Actin was used as a reference.

### 2.16. Statistical Analysis

All the above tests were repeated three times. GraphPad Prism 6.02 software was used for statistical analysis of data. Independent sample *t*-test was used to compare mean values of two samples. ANOVA was used to compare mean values in multiple groups. *P* ≤ 0.05 was considered statistically significant.

## 3. Results

### 3.1. SIRT6 Reduces the Destruction of the Femoral Head by Dexamethasone

**Figure 1:**
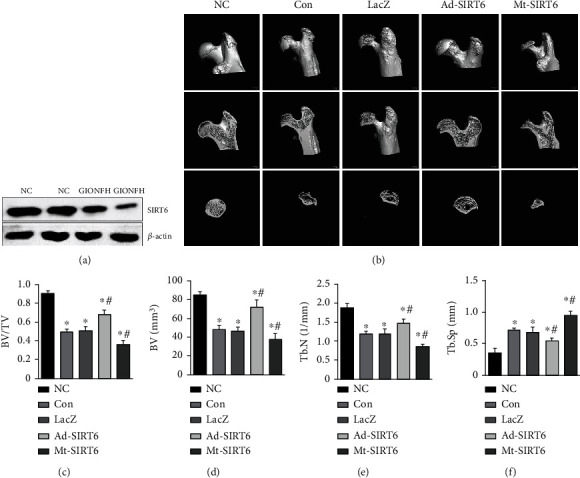
SIRT6 prevents disruption of femoral head microarchitecture. (a) The expression of SIRT6 was detected in the femoral head region of GIONFH by western blot. (b) Micro-CT scan image of the femoral head. (c–f) Bone parameters, including BV, BV/TV, Tb. N, and Tb. Sp. ^∗^*P* < 0.05 versus the NC group; ^#^*P* < 0.05 versus the model group.

In order to verify the changes of SIRT6 during the development of GIONFH, the changes of SIRT6 protein expression levels in the femoral head of normal and GIONFH model rats were compared by western blot. The expression of SIRT6 in the osteonecrosis area of the femoral head decreased in GIONFH rats (Figure 1(a)). According to the results of micro-CT scanning, it was found that dexamethasone caused the destruction of the bone surface structure of the femoral head in rats. It was rough, and there were a large number of cavities in the bone (Figure 1(b)). Bone mineral density and bone parameters BV and BV/TV decreased. After injection of SIRT6-overexpressing adenovirus, compared with the model control group, the surface of the femoral head was smooth, the structure of the femoral head was intact, the internal structure of the femoral head showed no significant abnormality, and BV and BV/TV were improved (Figures 1(c)–1(f)).

**Figure 2:**
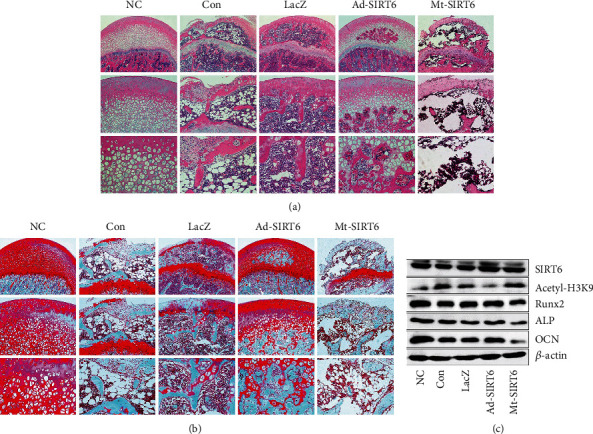
SIRT6 reduces the destruction of the femoral head structure by dexamethasone. (a) Representative images of HE staining of the femoral head. (b) Representative images of safranin-O staining of the femoral head. (c) Western blot was used to detect the expression of bone-related proteins.

The results showed that the cartilage layer of the femoral head became thinner, the bone arrangement was sparse and disordered, and the number of empty lacunae increased in the model group and adenovirus functional control group. These results could be reversed after SIRT6 overexpression treatment (Figures 2(a) and 2(b)). Relevant studies have found that SIRT6 mainly exerts various biological functions through its deacetylase function. SIRT6 mutation adenoviruses do not possess deacetylase activity and only retain ADP-ribosyltransferase activity. SIRT6 overexpression inhibited acetylation of histone H3 lysine 9 in the femoral head, whereas this effect was reversed after treatment with mutant SIRT6. In addition, further aggravation of femoral head destruction was observed in the SIRT6 mutation group. Meanwhile, the rat femoral head protein was extracted, and SIRT6 increased the expression levels of ALP, Runx2, and OCN and inhibited the expression of these proteins after SIRT6 mutation (Figure 2(c)).

### 3.2. SIRT6 Promotes Angiogenesis to Prevent ONFH by Inhibiting Ferroptosis

**Figure 3:**
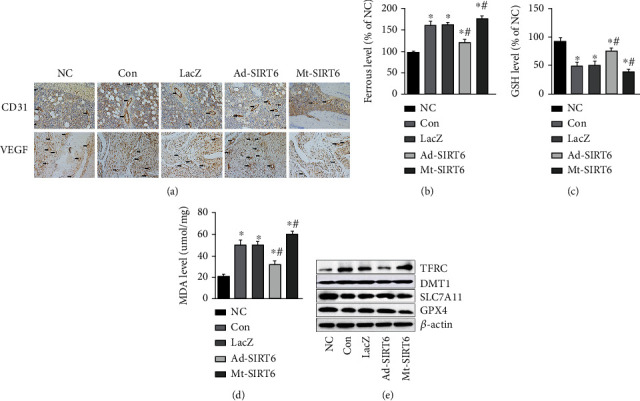
SIRT6 promotes angiogenesis to prevent ONFH by inhibiting ferroptosis. (a) Immunohistochemical staining for CD31 and VEGF-associated antigens and vascular density in the femoral head. (b) Serum ferrous iron levels in rats with GIONFH. (c) Serum GSH levels. (d) Serum lipid peroxidation product MDA levels. (e) Expression of ferritin and ferroptosis-related proteins in the femoral head. ^∗^*P* < 0.05 versus the NC group; ^#^*P* < 0.05 versus the model group.

In the early stage of GIONFH, intravascular pressure rises and vascular endothelium is mostly damaged. And related studies have found that SIRT6 has a role in protecting vascular endothelial cells and promoting angiogenesis [14, 15]. To verify the effect of SIRT6 on angiogenesis in rat femoral head, antibodies against CD31 and VEGF were selected for immunohistochemical staining (Figure 3(a)). The expression of CD31 and VEGF in the femoral head was decreased, the microvascular structure was destroyed, and there were few intact microvessels after dexamethasone administration. SIRT6 overexpression showed more CD31- and VEGF-positive cells, increased vascular density, and basically intact microvascular structure. What is more, the serum iron and ferritin levels of the model were higher than those of the normal control group (Figures 3(b)–3(d)). Protein was extracted from the femoral head (Figure 3(e)). A significant decrease in SLC7A11 and GPX4 expression was observed. And the contents of GSH and MDA also showed corresponding fluctuations. These results indicate that ferroptosis may have occurred in GIONFH.

### 3.3. SIRT6 Promotes Osteoblast Differentiation In Vitro

**Figure 4:**
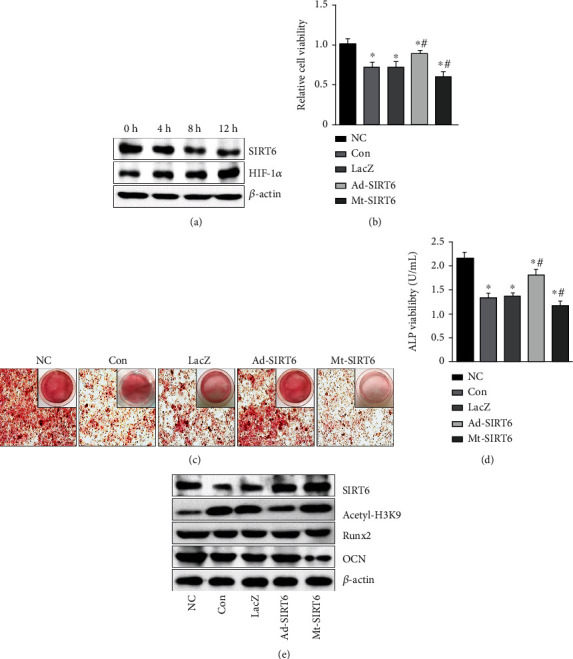
SIRT6 promoted osteogenic differentiation in vitro. (a) The expression level of SIRT6 in MC3T3-E1 cells after hypoxia was detected by western blot. (b) MTT assay was performed to examine the viability of MC3T3-E1 cells after hypoxia treatment. (c) The effect of SIRT6 on bone-related proteins in MC3T3-E1 cells after hypoxia. (d) ALP staining was used to detect ALP activity in MC3T3-E1 cells that induced osteogenic differentiation after 7 days. (e) The formation of mineralized nodules was detected by ARS staining in osteogenic differentiation induced 21 days in MC3T3-E1 cells. ^∗^*P* < 0.05 versus the NC group; ^#^*P* < 0.05 versus the model group.

Since the phenomenon of decreased SIRT6 expression levels in a rat model of GIONFH was found, whether SIRT6 was associated with osteoblast differentiation was further assessed *in vitro*. To mimic the in vitro ischemic environment, we subjected the cells to hypoxia. First, cells were subjected to hypoxia treatment for different periods of time to determine the most appropriate oxidative stress stimulation conditions. The expression level of SIRT6 decreased after hypoxia treatment in MC3T3-E1 cells, and the SIRT6 expression level became lower with prolonged hypoxia (Figure 4(a)). The viability of osteoblasts was decreased after hypoxia by MTT assay, partially restored after SIRT6 overexpression (Figures 4(b) and 4(c)). MC3T3-E1 cells treated with hypoxia had decreased ALP activity and decreased mineralized nodule formation compared with the normal group (Figure 4(d)). Alkaline phosphatase is a marker of the early stage of osteogenic differentiation, while the formation of calcium nodules is a marker of the later stage of osteogenic differentiation [16]. The expression of Runx2 and OCN was decreased in hypoxia-treated cells, indicating that hypoxia impaired osteoblast function (Figure 4(e)). The osteogenic function of MC3T3-E1 cells was further inhibited after SIRT6 gene mutation. SIRT6 overexpression protected the ALP activity and mineralization properties of MC3T3-E1 cells against hypoxia, and the expression of Runx2 and OCN was elevated. These results indicate that SIRT6 is essential for osteogenic differentiation.

### 3.4. SIRT6 Promotes Angiogenesis In Vitro

**Figure 5:**
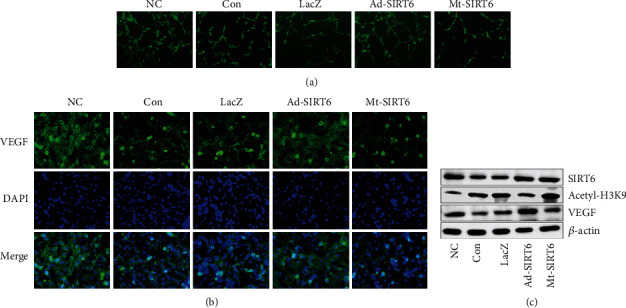
SIRT6 promotes angiogenesis. (a) SIRT6 promoted HUVEC to form a uterine-like structure as detected by tube formation assay in vitro. (b) Effect of SIRT6 on angiogenesis-related protein VEGF in HUVEC cells. (c) Immunofluorescence staining of HUVEC cells following interference with SIRT6.

The results showed that endothelial cells in the SIRT6 overexpression group differentiated into complete annular vessel-like structures, while vessel-like structures were incomplete or sparse in the SIRT6 mutation group (Figures 5(a) and 5(b)). Western blot results showed that hypoxia decreased the expression VEGF in HUVEC cells, and SIRT6 could improve the results (Figure 5(c)). Immunofluorescence showed the same results.

### 3.5. SIRT6 Inhibits Ferroptosis in GIONFH

**Figure 6:**
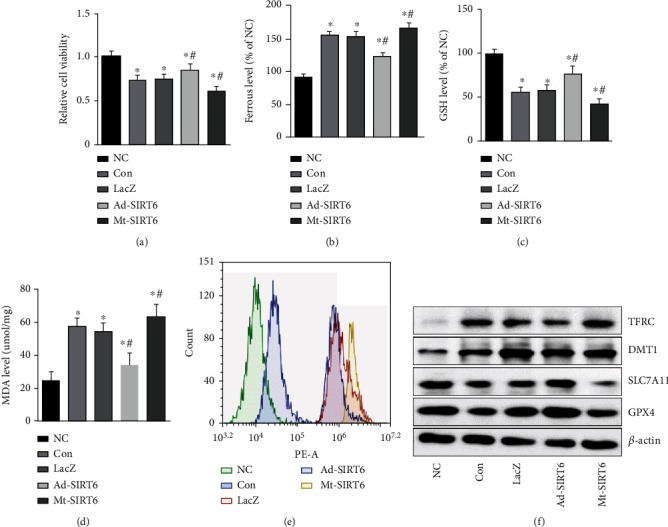
SIRT6 inhibits ferroptosis to prevent the development of osteonecrosis of the femoral head. (a) MTT assay was used to examine the viability of HUVEC cells after hypoxia. (b) Ferrous iron levels in HUVEC. (c) The content of GSH in HUVEC cells after interfering SIRT6. (d) The content of MDA in HUVEC cells. (e) Flow cytometry analysis of ROS levels. (f) The expression levels of ferroptosis-related proteins in HUVEC cells. ^∗^*P* < 0.05 versus the NC group; ^#^*P* < 0.05 versus the model group.

To further verify whether ferroptosis was activated in osteonecrosis of the femoral head, firstly, HUVEC cells were placed in a hypoxic environment and analyzed by MTT that hypoxia significantly reduced cell viability (Figure 6(a)). To analyze whether the decrease in cell viability was due to the occurrence of ferroptosis, we examined relevant measures of ferroptosis. The results showed that hypoxia could increase the consumption of glutathione and the content of malondialdehyde and increase the level of ROS in HUVEC cells (Figures 6(b)–6(e)). Hypoxia resulted in a decrease in the intracellular GPX4 and SLC7A11 expression levels. These results were exacerbated by the SIRT6 mutation group. However, SIRT6 overexpression could reverse the decrease of GPX4, SLC7A11, and GSH and reduce intracellular MDA and ROS accumulation (Figure 6(f)).

## 4. Discussion

This study verified the role of SIRT6 in GIONFH. This study found that dexamethasone downregulated SIRT6 expression, induced high ROS levels, and impaired osteogenesis and angiogenesis. Overexpression of SIRT6 can protect the activity of osteoblasts, promote osteoblastic differentiation, and reduce the damage of vascular endothelium, thereby preventing the occurrence of osteonecrosis of the femoral head.

Dexamethasone was applied to establish the GIONFH model in this study. This study found that the model group showed severe destruction of the bone structure in the subchondral bone area, necrosis of the surrounding bone marrow cells, and a large number of hollow lacunae. SIRT6 plays a key role in regulating bone formation and bone resorption. In this study, the effect of SIRT6 on GIONFH in rats was evaluated by micro-CT and histopathology and found that SIRT6 significantly improved the bone mass of the femoral head in glucocorticoid-induced rats, increased the number of trabecular, and decreased the separation of trabecular. New studies have found that Sirt6 Tg mice are resistant to the release of inflammatory factors and the progression of osteocyte death, bone resorption, and osteoarthritis after ischemic surgery, while osteoblast/osteocyte-specific SIRT6 knockout mice show severe bone loss and deformity [17]. SIRT6 could improve the viability of MC3T3-E1 cells in a femoral head necrosis model induced by hypoxia in vitro. Hypoxia also blocked osteoblast differentiation, while SIRT6 could improve the differentiation ability of osteoblasts injured by hypoxia, mainly reflected in increased ALP activity and increased number of calcified nodules. BMSCs from Sirt6 knockout mice had significantly lower alkaline phosphatase expression, reduced calcium nodule formation, and decreased osteogenic differentiation ability at the late stage of osteogenic differentiation, while Sirt6 knockout promoted the proliferation and differentiation of osteoclasts [18]. Western blot results also showed elevated expression of osteogenesis-related markers Runx2 and OCN after SIRT6 overexpression. The findings are consistent with previous findings that interference with SIRT6 can inhibit bone differentiation and affect osteogenesis [19, 20]. Therefore, these results suggest that SIRT6 may promote bone formation by elevating osteoblast viability and differentiation, thereby inhibiting osteonecrosis of the femoral head.

Massive use of glucocorticoid causes damage to vascular endothelial cells, triggering intravascular coagulation. Ischemia and osteonecrosis are the core pathological mechanisms of osteonecrosis of the femoral head. The expression of CD31, a key protein for angiogenesis, was downregulated. SIRT6 plays an important role in delaying endothelial cell senescence and protecting endothelial cell function [21, 22]. It was also demonstrated that SIRT6 could promote the generation of local blood vessels in the GIONFH model rat, and CD31 protein levels were markedly upregulated in bone tissue. Vascular endothelial cells, as the most important structural components in blood vessels, play a significant effect on maintaining the function of blood vessels and ensuring the normal blood supply of tissues. Hypoxia reduced the viability and angiogenic ability of HUVEC by MTT assay and tube formation assay in vitro. SIRT6 overexpression alleviated the damage of HUVEC induced by hypoxia and played a protective role. Previous studies have found that SIRT6 reduces the senescence of endothelial cells by reducing DNA damage and improving telomere function and maintains its ability to proliferate and form tubes in vitro [23]. CD31 is mainly expressed in vascular endothelial cells and is recognized as one of the classical markers of vascular endothelial cells [24]. VEGF is a hallmark regulator of angiogenesis. Hypoxia can downregulate the expression of CD31 and VEGF, while was upregulated after SIRT6 overexpression, which further indicated that SIRT6 could promote angiogenesis.

Ferroptosis is a new form of regulatory cell death. Abnormal intracellular iron metabolism, especially iron excess, leads to the continuous production of reactive oxygen species, which form and accumulate a large number of iron-dependent lipid peroxides in turn, which triggers ferroptosis. Several studies have shown that GC triggers ferroptosis [25]. First, in this study, we found that there was iron accumulation in GIONFH and that TFRC and DMT1 expression levels were increased. TFRC and DMT1 mediate the accumulation and transport of ferrous iron, leading to ferroptosis [26]. GPX4 inactivation is a feature of ferroptosis, and the activity of GPX4 depends on the supply of GSH, which is weakened by the rapid depletion of GSH. Hypoxia leads to inhibition of GPX4 activity in endothelial cells and that GSH concentration also decreases. However, SIRT6 increased not only GSH levels but also GPX4 expression levels. And the intracellular SLC7A11 expression level was also decreased after hypoxia treatment. SLC7A11 is a subunit of the XC-system and has a variety of functions, including the import of extracellular cysteine for glutathione biosynthesis, reactive oxygen species interpretation, and antioxidant activity [27]. SLC7A11 and GPX4 play a key role in preventing iron death mediated by lipid peroxidation. Inhibition of SLC7A11 and GPX4 interrupts intracellular GSH metabolism and promotes lipid peroxidation and subsequent ferroptosis [28]. Ferroptosis is also closely related to lipid peroxidation as well as the increase of reactive oxygen species. In addition, MDA is a marker of lipid peroxidation products [29]. Upregulation of SIRT6 can decrease the levels of intracellular MDA and ROS in vascular endothelial cells. However, current studies on the role of SIRT6 in ferroptosis are still limited. Recently, it has been demonstrated that sodium hydrogen sulfide can enhance SIRT6 expression and inhibit ferroptosis in the frontal cortex of diabetic mice [30]. Consistent with their findings, SIRT6 overexpression inhibited ferroptosis in vascular endothelial cells in this study. These results demonstrate that overexpression of SIRT6 can reverse hypoxia-induced ferroptosis.

## 5. Conclusion

In summary, SIRT6 can inhibit the occurrence of ferroptosis, reduce the damage of vascular endothelium, and promote osteogenic differentiation, thereby preventing the occurrence of osteonecrosis of the femoral head. Our study provides a theoretical basis for SIRT6 as a potential target for GIONFH therapy.

## Data Availability

The datasets during the current study are available from the corresponding authors on reasonable request.
